# Twelve-year follow-up study after endoscopic sinus surgery in patients with chronic rhinosinusitis with nasal polyposis

**DOI:** 10.1186/s13601-019-0269-4

**Published:** 2019-06-14

**Authors:** Lien Calus, Nicholas Van Bruaene, Cedric Bosteels, Sarah Dejonckheere, Thibaut Van Zele, Gabrielle Holtappels, Claus Bachert, Philippe Gevaert

**Affiliations:** 1 Upper Airways Research Laboratory, Department of Otorhinolaryngology, Ghent University Hospital, De Pintelaan 185, 9000 Ghent, Belgium; 2 Division of ENT Diseases, Clintec, Karolinska Institutet, Stockholm, Sweden

**Keywords:** Nasal polyps, Sinusitis, Paranasal sinus diseases, Nasal surgical procedures, Paranasal sinuses

## Abstract

**Background:**

Chronic rhinosinusitis with nasal polyposis (CRSwNP) is a therapeutic challenge because of the high recurrence rate. Surgical intervention should be considered in patients who fail to improve after medical treatment. We monitored recurrence and revision surgery over 12 years after endoscopic sinus surgery in CRSwNP patients.

**Methods:**

In this prospective cohort study, 47 patients with CRSwNP, who underwent primary or revision extended endoscopic sinus surgery, were followed. Clinical symptoms and total nasal endoscopic polyp score were evaluated before, 6 years and 12 years after surgery.

**Results:**

Twelve years after surgery, 38 out of 47 patients (80.9%) were available for examination. There still was a significantly better symptom score and total nasal endoscopic polyp score compared to before surgery (P < 0.001). Within the 12-year follow-up period, 30 out of 38 patients developed recurrent nasal polyps, of which 14 patients underwent additional revision surgery. Comorbid allergic sensitization and tissue IL-5 levels were found to be significant predictors for the need of revision surgery.

**Conclusions:**

This long-term cohort study, investigating the outcome after surgery in CRSwNP, showed that, despite the low number of patients, 78.9% of patients with CRSwNP were subject to recurrence of the disease and 36.8% to revision surgery over a 12-year period.

**Electronic supplementary material:**

The online version of this article (10.1186/s13601-019-0269-4) contains supplementary material, which is available to authorized users.

## Background

The treatment of chronic rhinosinusitis with nasal polyposis (CRSwNP) is a therapeutic challenge for ENT-specialists, pulmonologists, and allergists. This inflammatory disease of the nose and paranasal sinuses with nasal polyps (NP) accounts for substantial health care expenditures in terms of office visits, antibiotic prescriptions, lost workdays and missed school days [1]. CRSwNP is frequently associated with asthma and intolerance for aspirin or non-steroidal anti-inflammatory drugs [called aspirin exacerbated respiratory disease (AERD)] [2]. This difficult-to-treat group suffers from a more severe upper and lower airway disease, reflected by high NP recurrence and frequent need of endoscopic sinus surgery (ESS) [3, 4]. The relationship between CRSwNP and allergy remains incompletely defined and there is no causal association proven [2].

Overall it has been stated that CRSwNP in Caucasians is an eosinophilic T helper (Th) 2 biased inflammation with high levels of local interleukin-5 (IL-5) and immunoglobulin E (IgE) [5]. Some progress has been made in elucidating the underlying pathomechanism of CRSwNP. For example, the role of *Staphylococcus aureus* as an important disease modifier has been demonstrated [6].

Due to the high recurrence rate, the goal of the treatment of nasal polyps is to achieve and maintain clinical control. In other words, patients should not have symptoms, or the symptoms should not be bothersome, if possible combined with a healthy or almost healthy mucosa and only local treatment [2]. The medical treatment in CRSwNP is based on topical or intranasal corticosteroids, systemic or oral corticosteroid and antibiotics [2].

Over time, ESS evolved to be the treatment of choice in CRSwNP, when conservative treatment failed. When considering the efficacy of surgery in CRSwNP, few randomized controlled trials are available but the studies have demonstrated that sinus surgery in patients with nasal polyps can result in a prolonged reduction of nasal symptoms and an improvement of quality of life [7–11]. However, regardless of the surgical technique applied, a fair number of patients will present with recurrent CRSwNP disease at some point in time. Disease recurrence ranged from 4 to 60% in CRSwNP with a median of 20% across all studies reviewed over maximum 2 years [8, 12]. When NP recurrence occurs patients sometimes undergo revision surgery. The revision surgery rate varies between 4 and 27% with follow-up periods varying between 12 and 60 months [7, 12].

Different studies have examined prognostic factors, like tissue eosinophilia, affecting the success of endoscopic sinus surgery [13]. Surgery removes the disease burden but also increases the efficacy of postoperative medical treatment. Prolonged postoperative medical treatment with topical corticosteroid sprays would appear to improve outcomes after ESS in CRSwNP [2].

The impact of different comorbidities has also extensively been investigated. Studies contradict each other about the influence of allergic sensitization on the outcome of ESS [12]. However, different studies have observed that CRSwNP patients with asthma or AERD have higher recurrence rates [4, 12, 14, 15].

Though, there are a lot of studies investigating the outcomes after ESS, the follow-up is in most studies short (12 months) and retrospective. Some scarce studies performed a longer follow–up between 5 and 20 years, but these studies use a wide variation of surgical techniques [4, 15, 16]. There is also a lack of knowledge of risk factors that might increase the likelihood of NP recurrence and revision surgery. Therefore, we performed a prospective cohort study in CRSwNP after ESS over a 12-year follow-up period. To minimize confounding factors, one surgeon performed a standardized surgical procedure in all patients. Further, at baseline all patients were extensively characterised based on clinical characteristics, comorbidities and inflammatory factors in serum, nasal secretions and tissue. The goal of our study was to look at NP recurrence and the need of revision surgery over a 12-year follow-up period after surgery.

## Methods

### Patients and study design

The patient population of this 12-year prospective cohort study covers 47 patients, 18 years or older, with CRSwNP diagnosed based on history, clinical examination, nasal endoscopy and CT scan, following the current EPOS guidelines [2]. All patients underwent extended ESS for nasal polyposis at the department of Otorhinolaryngology of the Ghent University, Belgium, between 1998 and 2000. The description of the surgical technique can be found in this article’s Additional file 1.

Before surgery, all patients underwent skin prick testing for common aero-allergens to diagnose allergic sensitization [17]. The presence of asthma was investigated by a lung physician [18] and the diagnosis of aspirin intolerance was primarily based on the clinical picture, namely the presence of asthma, nausea, erythema or other complaints shortly after ingestion of aspirin or NSAIDs. The exclusion criteria were pregnancy, cystic fibrosis, primary ciliary dysfunction, Kartagener syndrome, parasitic infection, and fungal infections.

In the scope of this study, three visits at the ENT-department were organized: pre-operatively, approximately 6 and 12 years after initial surgery.

The ethics committee of Ghent University Hospital approved the study and all patients provided written informed consent before participation in the study.

### Outcome measures

As primary outcome NP recurrence and the need of revision surgery were examined. The NP recurrence over 12 years’ time was defined as a nasal polyp score greater than 0 at one of the two follow-up visits or a history of revision surgery for recurrent NP during the 12-year follow-up period. The revision surgery rate is based on the exact surgical dates collected at each visit and by revision of medical records.

At each time point the following data were collected: demographics, comorbidities, symptom score (nasal obstruction, rhinorrhea, smell disturbance, sneezing, headache and eye symptoms), total nasal endoscopic polyp score (Davos score), history of previous surgery, medication use, general therapeutic response and EPOS control test. More detailed information can be found in this article’s Additional file 1.

### Collection of tissue, serum and nasal secretion

Nasal polyp tissue, nasal secretion and serum were collected and investigated for different inflammatory markers [IL-5, IL-5Rα, TGF-β1, MPO, IL-18, ECP, total IgE and specific IgE antibodies against *S*. *aureus* enterotoxins (SAE-IgE)]. More detailed information about the used techniques can be found in this article’s Additional file 1.

### Statistics

Data are expressed as absolute numbers and percentages for categorical and as median and interquartile range (IQR) for continuous variables. Further details about statistics can be found in this article’s Additional file 1.

## Results

### Patient characteristics

Forty-seven CRSwNP patients were included prior to ESS. Table 1 shows the clinical baseline characteristics of these 47 patients. A follow-up was performed over 12 years and in 2006 and 2012 patients were invited for an extensive follow up visit. After 12 years the response rate was 79% (38 out of 47), of which 19 patients underwent in 2000 primary surgery and 19 underwent revision surgery. We show in Table 2 the clinical baseline characteristics of both groups and we observe at baseline a significant higher amount of IL-5, IL-5Ra and ECP in patients undergoing revision surgery compared to primary surgery. Over the 12-year period 3 patients deceased, 4 patients could not be traced, and 2 patients were not prepared to participate.

**Table 1:** Baseline clinical characteristics in the group included in 2000 (N = 47)

*Baseline clinical characteristics (N = 47)*	
Clinical characteristics	
Men/women, N/N (%/%)	33/14 (70.2/29.8)
Age (y), median (IQR)	49 (37–58)
Primary/revision ESS in 2000, N/N (%/%)	22/25 (46.8/53.2)
Comorbidity	
Allergic sensitization, N (%)	24 (51.1)
Asthma, N (%)	18 (38.3)
AERD, N (%)	11 (23.4)
Total NP score, median (IQR)	4 (3–6)
Total symptom score, median (IQR)	8 (7–11)
Tissue biomarkers, median (IQR)	
IL-5 (pg/ml)	133.24 (43.00–338.58)
Detectable IL-5, N (%)	31 (66.0%)
IL-5Rα (pg/ml)	5003.01 (1765.61–21,069.23)
TGF-β1 (pg/ml)	9534.22 (7457.03–20,760.85)
MPO (ng/ml)	6878.56 (2805.72–14,874.40)
IL-18 (pg/ml)	15,373.60 (6738.21–22,019.14)
ECP (mg/l)	7.46 (1.84–14.87)
Total IgE (kU/l)	432.30 (146.30–1155.90)
IgE Grass mix 1 (kU/l)	3.88 (0.00–5.78)
IgE Tree mix 9 (kU/l)	4.30 (0.00–8.03)
IgE House dust mite mix 2 (kU/l)	4.08 (0.00–8.03)
Detectable IgE to SAE, N (%)	18 (39.1)
Nasal secretion biomarkers, median (IQR)	
IL-5 (pg/ml)	30.00 (30.00–131.21)
IL-5Ra (pg/ml)	1135.84 (497.57–5357.62)
ECP (mg/l)	0.53 (0.25–1.16)
IgE (kU/l)	283.44 (109.38–440.85)
Serum biomarkers, median (IQR)	
IL-5 (pg/ml)	BDL
IL-5Ra (pg/ml)	414.90 (297.00–744.70)
ECP (µg/l)	26.10 (21.00–40.00)
IgE (kU/l)	157.0 (48.5–278.0)

*N* number, *IQR* interquartile range, *BDL* below detection level

**Table 2:** Baseline clinical characteristics in patients, who were followed for 12 year (N = 38), with primary or revision ESS at baseline

	Primary ESS	Revision ESS	*P* value
	N = 19	N = 19	
*Baseline clinical characteristics (N = 38)*			
Clinical characteristics			
Men/women, N/N (%/%)	13/6 (68/32)	12/7 (63/37)	1.00
Age (y), median (IQR)	44 (31–54)	47 (37–53)	0.863
Comorbidity			
Allergic sensitizaion, N (%)	10 (53)	10 (53)	1.00
Asthma, N (%)	5 (26)	10 (53)	0.184
AERD, N (%)	3 (16)	7 (37)	0.269
Total NP score, median (IQR)	4 (3–5)	4 (4–6)	0.644
Total symptom score, median (IQR)	8 (7–12)	9 (8–12)	0.443
Tissue biomarkers, median (IQR)			
IL-5 (pg/ml)	86.02 (43.00–237.87)	228.14 (133.24–484.44)	*<* *0.05*
Detectable IL-5, N (%)	10 (53)	16 (84)	0.079
IL-5Rα (pg/ml)	2704.74 (1116.04–13,162.88)	15,383.50 (4036.26–28,350.59)	*<* *0.05*
TGF-β1 (pg/ml)	8934.18 (7356.03–15,825.42)	12,769.26 (7562.52–27,256.90)	0.477
MPO (ng/ml)	5251.91 (2634.00–11,020.06)	9255.17 (4602.93–19,868.30)	0.061
IL-18 (pg/ml)	14,982.30 (7761.63–25,611.80)	15,051.30 (4698.93–19,633.68)	0.354
ECP (mg/l)	5.71 (0.86–12.93)	13.48 (4.75–28.46)	*<* *0.05*
Total IgE (kU/l)	322.95 (146.30–1348.16)	432.30 (251.83–1156.39)	0.639
Detectable IgE to SAE, N (%)	8 (42)	8 (42)	1.00
Nasal secretion biomarkers, median (IQR)			
IL-5 (pg/ml)	30.00 (30.00–109.22)	94.81 (30.00–169.86)	0.285
IL-5Rα (pg/ml)	667.55 (439.40–791.74)	2897.67 (562.93–10,030.07)	0.076
ECP (mg/l)	0.36 (0.12–0.79)	0.54 (0.32–3.57)	0.100
IgE (kU/l)	174.15 (40.49–312.94)	324.35 (100.14–662.78)	0.394
Serum biomarkers, median (IQR)			
IL-5 (pg/ml)	BDL	BDL	ND
IL-5Ra (pg/ml)	334.60 (235.60–710.05)	414.90 (364.30–866.60)	0.471
ECP (µg/l)	26.10 (21.60–45.15)	23.90 (19.75–189.50)	0.601
IgE (kU/l)	217.00 (51.15–715.00)	50.20 (29.35–189.50)	0.110

Italic values indicate significance of *P* value (*P* < 0.05)

*N* number, *IQR* interquartile range, *BDL* below detection level, *ND* not done

### Nasal obstruction and smell disturbance improved 12 years after surgery

**Fig. 1:**
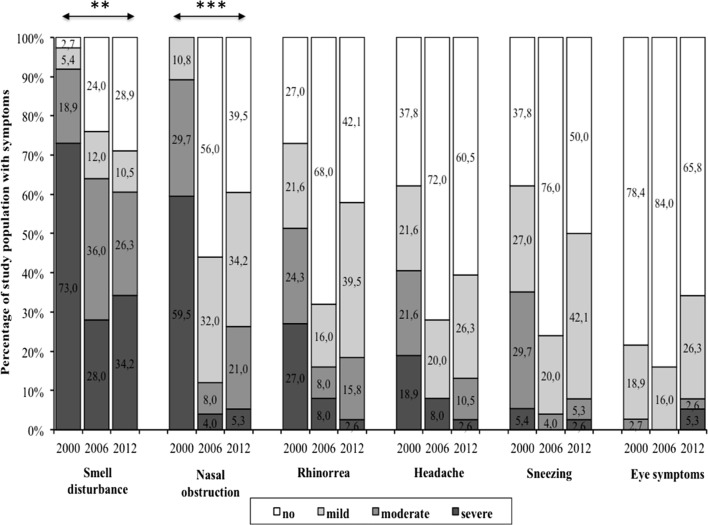
Symptoms, expressed as no, mild, moderate or severe are shown as the percentage of study population that experience complaints with the respective severity, at each time point (2000 N = 47, 2006 N = 27 and 2012 N = 24). The percentages are marked in the bars. **P < 0.01; ***P < 0.001

Smell disturbance and nasal obstruction were the most predominant symptoms pre-operatively, as shown in Fig. 1. These symptoms are scored bothersome, i.e. moderate to severe, in 91.9% and 89.2% patients respectively for smell disturbance and nasal obstruction. Twelve years after surgery the smell disturbances (P < 0.01) and the nasal obstruction (P < 0.001) were still significantly improved compared to baseline (see Fig. 1). Rhinorrhoea and headache, two other symptoms mentioned in the EPOS definition, were also pre-operatively reported as bothersome in 51.3% and 40.5% of the patients respectively. After 12 years those complaints decreased to respectively 18.4% and 13.1%. Compared to the aforementioned symptoms, patients were less troubled by sneezing and eye symptoms pre-operatively and during follow-up.

### Nasal polyps are absent in 40% of the patients 12 years after surgery

**Fig. 2:**
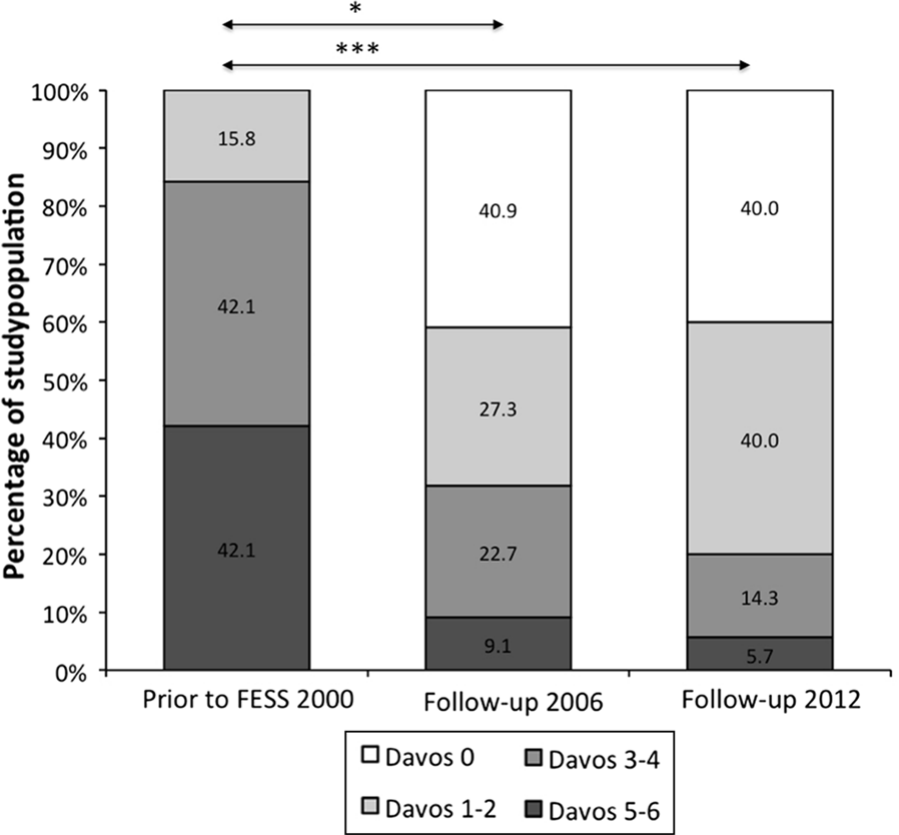
The total nasal endoscopic polyp score expressed as Davos score between 0 and 6. The total nasal endoscopic polyp score is divided in four groups and the percentage of study objects with a certain Davos score are shown at the three time points (2000 N = 47, 2006 N = 27 and 2012 N = 24). *P < 0.05; ***P < 0.001

In 2000, prior to ESS, all patients had considerable nasal polyps (Fig. 2). Compared to the NP score prior to ESS, the NP score was significantly decreased in 2006 (P < 0.05) and in 2012 (P < 0.001). Twelve years after ESS, endoscopic examination of the nasal cavity showed in 40% of the patients no nasal polyps. However, this percentage also includes patients that underwent revision surgery during the 12-year follow-up study. This percentage thus underestimates the presence on nasal polyps over the 12-year period.

### 78.9% of the patients ‘ever’ experienced recurrence of nasal polyps

**Fig. 3:**
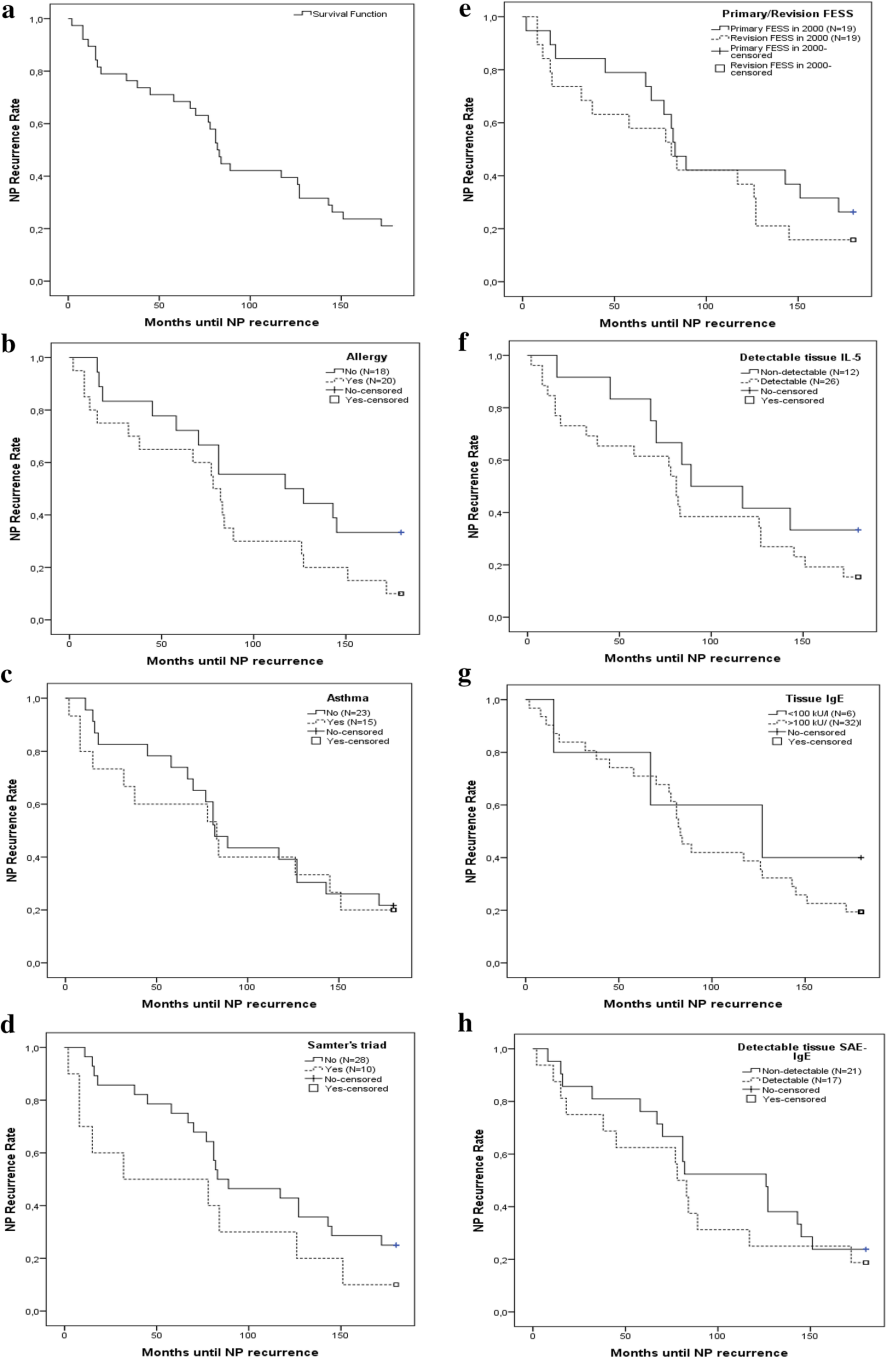
The performance of NP recurrence expressed over time as a Kaplan–Meier curve (**a**). **b** The Kaplan–Meier curve in patients with or without allergic sensitization, **c** in patients with or without asthma, **d** in patients and with or without AERD and **e** in patients with primary or revision surgery. The Kaplan–Meier curves for NP recurrence rate based on the detection of IL-5 (**f**), presence of IgE higher than 100 kU/l (**g**) and detection of SAE IgE (**h**) in tissue

Thirty out of 38 CRSwNP patients or 78.9% developed NP recurrence at a certain time point during 12 years of follow-up. Figure 3a shows the development of recurrence over time.

**Fig. 4:**
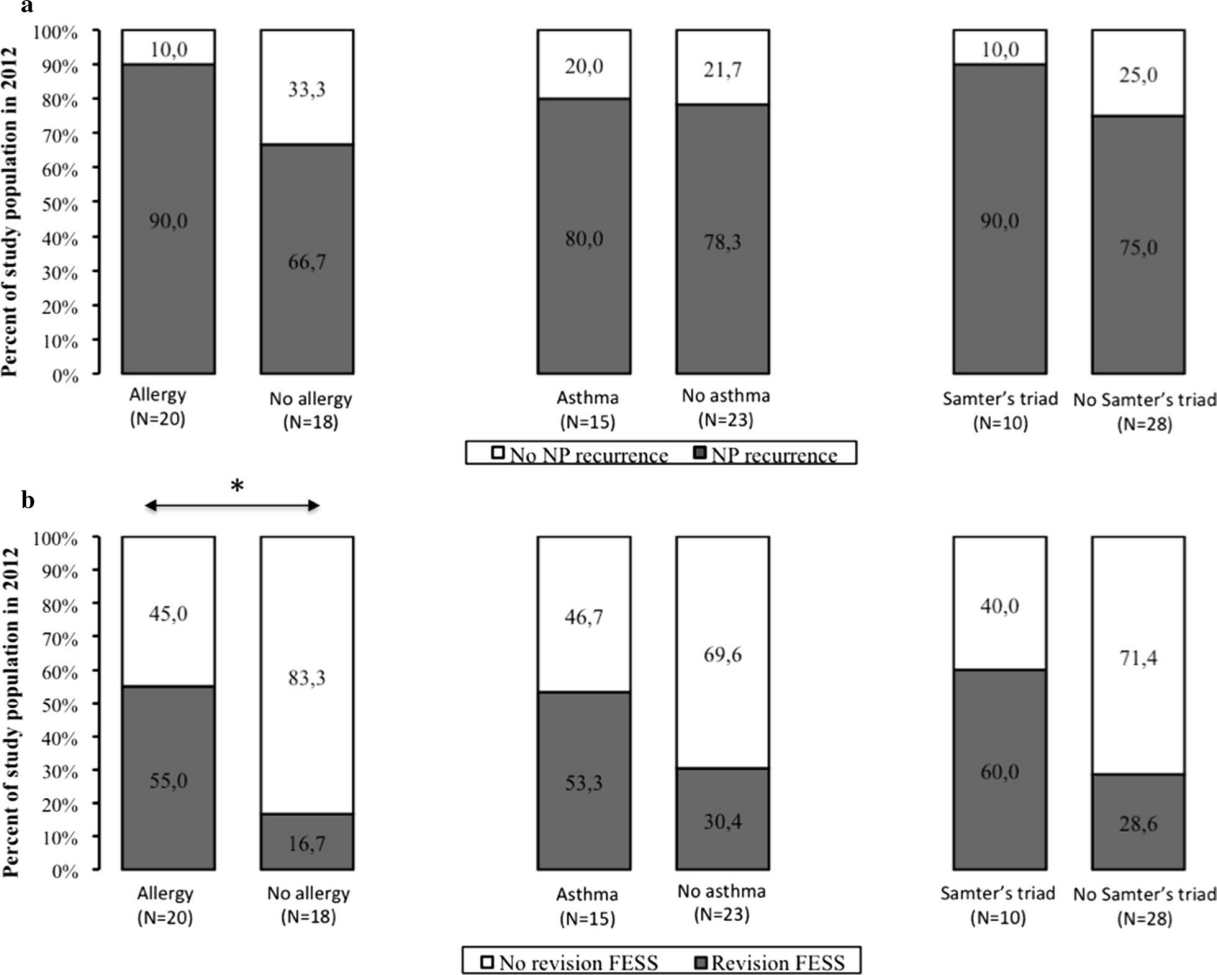
The percentage of NP recurrence, divided by the presence or absence of allergic sensitization, asthma and AERD are shown in **a**. Panel **b** shows the percentage of patients with revision surgery, divided for the presence or absence of allergic sensitzation, asthma and AERD. *P < 0.05

Comparing the ‘NP recurrence’ (N = 30) and ‘no recurrence’ group (N = 8; see Additional file 1: Table S1), we identified that 60% of the NP recurrence group had comorbid allergic sensitization however not significant different with 25% in the ‘no recurrence’ group (P = 0.117). Figure 4a shows the contribution of asthma and AERD. In the NP recurrence group 80.0% expressed asthma and 90.0% AERD compared to 78.3% asthma and 75.0% AERD in the non-recurrence group (Fig. 4a). The logistic regression showed a trend towards increased risk of NP recurrence in allergic patients (OR 4.5, 95% CI 0.78 to 26.1, P = 0.094) however not significant. For asthmatic patients (OR 1.1, 95% CI 0.22 to 5.5, P = 0.898) and patients with AERD (OR 3.0, 95% CI 0.32 to 28, P = 0.336) the risk is less obvious. This is confirmed by the Kaplan–Meier graphs (allergy Fig. 3b P = 0.093; asthma Fig. 3c P = 0.738; AERD Fig. 3d P = 0.136).

Looking at the tissue markers there was no statistical difference between tissue IL-5, ECP and IgE (see Table S1 in Additional file 1).

The non-significant influence of tissue IL-5 (Fig. 3f; P = 0.233), IgE (Fig. 3g; P = 0.446) and SAE-IgE (Fig. 3h; P = 0.459) on the development of NP recurrence over time is shown in the Kaplan–Meier figures.

### 36.8% of the patients underwent revision surgery

Fourteen out of 38 or 36.8% of the CRSwNP patients had a need of revision surgery in the 12-year follow-up period. Consequently over 12-year follow-up the patient group can be divided in three groups: no recurrence (21.05%), NP recurrence but no revision ESS (42.11%) and NP recurrence with revision ESS (36.84%).

**Fig. 5:**
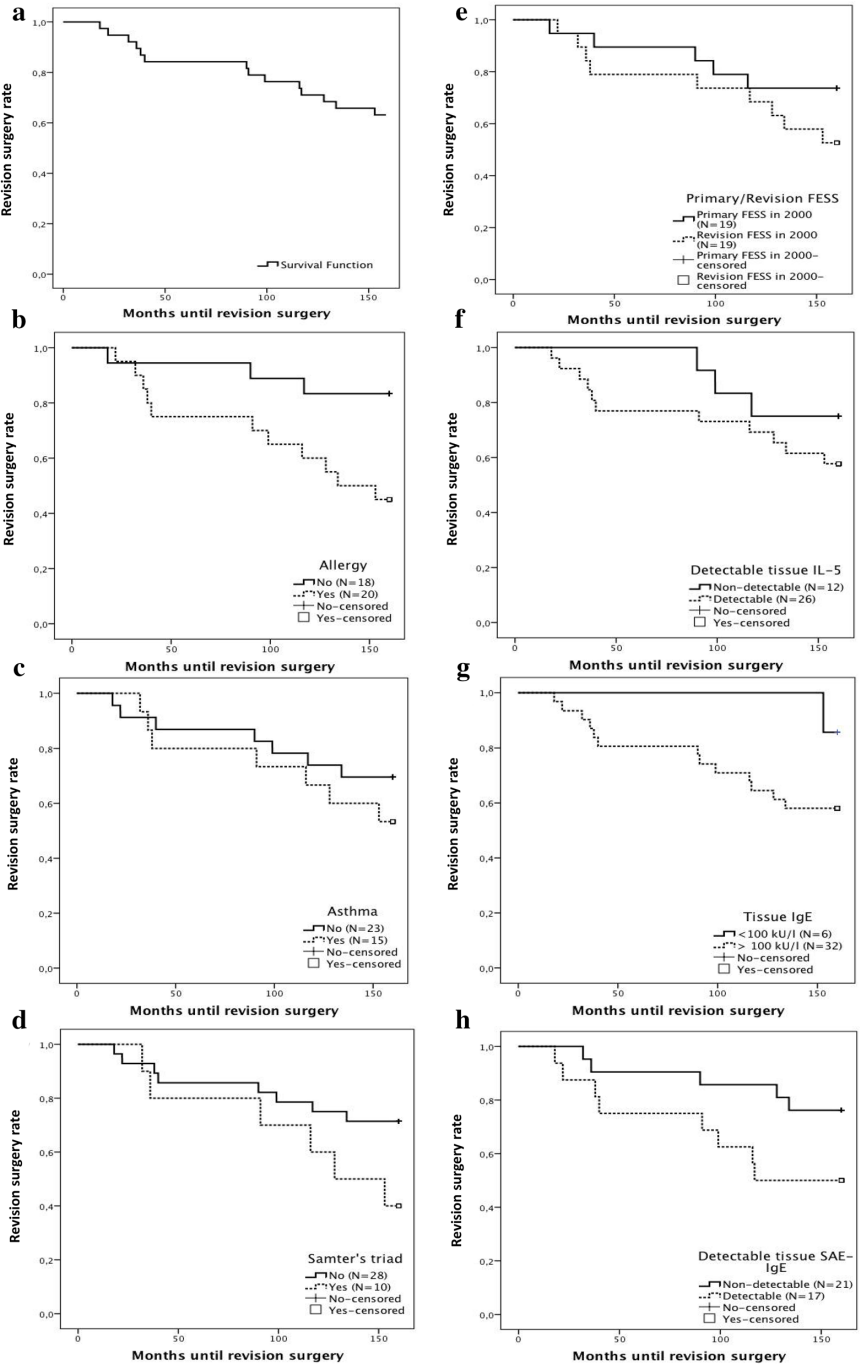
The performance of revision surgery expressed over time as a Kaplan–Meier curve (**a**). **b** The Kaplan–Meier curve for revision surgery rates in patients with or without allergic sensitization, **c** in patients with or without asthma, **d** in patients and with or without AERD and **e** in patients with primary or revision surgery. The Kaplan–Meier curves for revision surgery rate based on the detection of IL-5 (**f**), presence of IgE higher than 100 kU/l (**g**) and detection of SAE IgE (**h**) in tissue

Figure 5a illustrates Kaplan–Meier survival analysis for revision surgery within the 12-year follow-up based on the date of revision surgery, with an overall ‘surgery-free rate’ of 84.2% (32/38) at 6 years and 63.2% (24/38) at 12-year follow-up. The time to revision ESS ranged from 18 to 153 months, with a median of 91 months.

The possible contribution of allergic sensitization, asthma and AERD to the risk of revision surgery was investigated and only allergic sensitization could be identified as a significant predictor for the need of revision surgery during follow-up (OR 6.1, 95% CI 1.3 to 28, P < 0.05 in allergic sensitization). The percentage patients who underwent revision surgery in asthmatics (53.3%) and patients with AERD (60.0%) was higher compared to patients without asthma (30.4%) or AERD (28.6%), however there was no statistical difference of revision surgery in the presence or absence of asthma and AERD (Fig. 4b).

Moreover, the survival curve showed that the ‘surgery-free survival rate’ was significantly longer in non-allergic patients compared to allergic patients (Fig. 5b; P < 0.05). The ‘surgery-free survival rate’ for revision ESS did not significantly differ in patients with primary or revision surgery (Fig. 5e; P = 0.220), in patients with or without asthma (Fig. 5c; P = 0.350) and in patients with and without AERD (Fig. 5d; P = 0.098).

Comparing the baseline characteristics, tissue IL-5 is significantly higher in patients undergoing revision surgery [360.84 pg/ml (103.92–521.84)] compared to patients who did not need revision surgery (with and without NP recurrence; 112.94 pg/ml (43.00–228.88); P < 0.05; see Additional file 1: Table S2). Importantly, we identified tissue IL-5 as a significant predictor for revision surgery over the follow-up period (OR 1.004, 95% CI 1.001 to 1.008, P < 0.05). This implicates that the higher the IL-5 levels pre-operatively the higher the risk for a revision surgery.

Additionally, tissue IgE (Fig. 5g; P = 0.166) or SAE-IgE (Fig. 5h; P = 0.294) was not a predictive factor for revision surgery.

### Medication use is an important cornerstone of treatment

The use of medication did not significantly differ between the 3 moments of contact, although asthma medication use tended to be higher during follow-up (Additional file 1: Fig. S1). In 2006 64.0% of patients and in 2012 52.6% of patients used intranasal corticosteroids as an ongoing treatment, compared to 57.9% pre-operatively. Additionally, 5 patients received during the 12-year follow-up period monoclonal antibodies in double blind randomized controlled trials (3 patients received anti-IL-5 [22], 1 patient anti-IgE [25] and 1 patient both). Four of these patients demonstrated a clinical response to the treatment, and four patients required surgery after termination of treatment. The Kaplan–Meier curves were generated for revision surgery and revision surgery plus biologics but did not show any difference.

### General therapeutic relief after ESS is good

When inquiring general therapeutic relief after the ESS in 2000 over the 12-year period, 8 out of the 38 (21.1%) patients reported a complete, 36.8% a marked, 26.3% a moderate and 13.2% a slight relief over time.

The EPOS control test was performed retrospectively and showed that initially all patients (97.4%) were uncontrolled before surgery. Six years after surgery 40% was uncontrolled and 44% was partially controlled based on symptoms and medication needed. Only 16% was controlled 6 years after surgery. At the endpoint, 12 years after inclusion, 47.4% was uncontrolled, 26.30% was partially controlled and 26.30% was controlled.

We asked patients if they would do the ESS again 12 years ago with the knowledge they have today. Thirty-six (94.7%) out of the 38 patients answered ‘Yes’. Two patients (5.3%) answered ‘No’, of which one was due to postoperative bleeding.

## Discussion

CRSwNP is a recalcitrant condition, which needs ongoing treatment. This long-term prospective study investigated the outcome after ESS in patients suffering from CRSwNP over a 12-year period. We showed that 78.9% of the patients with CRSwNP were subject to recurrence of the disease and 36.8% to revision surgery over a 12-year period. This study differed from previous follow-up studies by extensive characterization of the patients based on clinical characteristics and on local inflammatory parameters, like IL-5, IgE, SAE-IgE. This gave the opportunity to identify comorbid allergic sensitization and local IL-5 as predictive risk factors for the need of revision surgery.

The presence of nasal polyps induces nasal obstruction and smell disturbance, which are considered the most abundant symptoms [2]. This study showed that 12 years after ESS a clinical significant improvement is observed based on subjective symptoms and objective nasal endoscopic polyp score. Thus, ESS may contribute to the long-term alleviation of the subjective and objective burden of CRSwNP, which is in line with the findings in short term [8, 10, 16]. It is important to notice that our results were influenced by revision surgery, which was performed in certain patients over the 12-year follow up period and also by long-term post-operative medical treatment, like nasal corticosteroids.

Our results showed that a substantial part of the patients (78.9%) develop NP recurrence over time. In our study the NP recurrence rate after surgery was based on objective endoscopic visualization of polyps in the nasal cavity. The NP recurrence rates reported in previous research varied substantially for several reasons [4, 8, 19], including the short term duration of follow-up, different post-operative medical treatments, variations in the surgical techniques and different definitions of NP recurrence (endoscopy, imaging, symptoms, etc.). Our study was unique due to the prospective and long-term approach. In this study, one surgeon performed a standardized surgical procedure at the university hospital of Ghent; thereby the influence of experience and technique was minimized [8, 20, 21].

Patients included in this study are extensively charactarised by comorbid allergic sensitzation, asthma, aspirin hypersensitivity, IL-5 and ECP amounts in tissue. Unfortunately, due to the limited number of patients of this study, we could not identify significant risk factors for NP recurrence.

Our data indicated that revision surgery was needed in 36.8% of the CRSwNP patients over the 12-year follow-up. The majority of studies investigating revision surgery have expressed relapse rates as a point estimate during a mean duration of follow-up [7, 12]. Kaplan- Meier survival analysis enables estimation of revision surgery rates across time and therefore our median time to revision ESS was 91 months or almost 8 years. Our study identified different significant risk factors for the need of revision surgery like comorbid allergic sensitization; namely allergic patients underwent a revision surgery sooner than non-allergic patients. This strengthens the importance of allergic sensitization diagnosed pre-operatively by skin prick test as a possible predictive factor for a poor outcome. In literature, asthma and AERD could be withheld as determinants for revision surgery [14, 15].

The pathophysiology of CRSwNP is characterized by high local IL-5 and IgE levels [5]. In the current study, tissue IL-5 levels were identified as a positive predictive factor for the need of revision ESS. Patients with detectable IL-5 in tissue have an increased risk for the need of revision surgery over time. Our study proved that this cytokine, important in the pathophysiology of CRSwNP, also plays a pivotal role in the prognosis after ESS in CRSwNP patients. Currently, there is a therapeutic option available, namely, Mepolizumab or anti-IL-5, which has been proven effective in CRSwNP [22]. Perhaps in the future this treatment can be proposed to prevent NP recurrence and the need of revision surgery.

Local polyclonal tissue IgE is also a cardinal feature of the local inflammation present in CRSwNP [6]. Recent evidence has accumulated, suggesting that *S*. *aureus* enterotoxins induce a local polyclonal IgE formation combined with an increased risk for developing asthma [23, 24]. Local IgE are is higher revision surgery. Currently a targeted treatment against IgE, Omalizumab (anti-IgE), has proven favourable effects in CRSwNP [25]. IgE can therefore be used as a prognostic and therapeutic factor in CRSwNP.

An important remark should be made, the clinical and inflammatory profile of the patients at baseline differed. We believe that this could interfere with the results. We need to acknowledge that at baseline patients who already had surgery before 2000 were included. This group of revision surgery at baseline is believed to have a more severe local eosinophilic inflammation with high IL-5 and ECP.

In the future a similar study with a greater number of patients should be performed to confirm our results. The number of patients in this study is limited but this study shows the extent of recurrence and revision surgery in CRSwNP.

The study might be biased by the post-operative treatment. Generally, our patients were treated following the EPOS guidelines, i.e. rinsing with physiologic water, nasal corticoids in spray and drops, occasionally doses of doxycycline or oral corticosteroids. Additionally, 5 patients received during the 12-year follow-up period monoclonal antibodies in double blind randomized controlled trials (3 patients received anti-IL-5 [22], 1 patient anti-IgE [25] and 1 patient both). The clinical response of 4 patients emphasises the importance of new monoclonal treatment options next to surgery [26].

Finally, our study proved that the vast majority of the patients experienced ESS as a beneficial procedure that improved their general wellbeing. In some patients NP recurrence was diagnosed by nasal endoscopy during the 12-year follow-up period, but they did not (yet) decide to perform a revision surgery, for example based on minimal symptoms, on attempts of conservative management or on time required to schedule a surgery. This strengthens the importance of other subjective factors in the decision for revision surgery. This is in contrast to NP recurrence, which is an inflammatory pathophysiological mechanism, independent of subjective patient-related factors.

As conclusion, CRSwNP is a chronic condition with a high recurrence and revision surgery rate over 12 years follow-up. Sinus surgery for CRSwNP patients should not be the only treatment option but rather be a modality used to manage patients to remove the disease burden and increase the efficacy of post-operative medical therapy. Regular follow-up is important for this chronic disease and chronic treatment with topical corticosteroids should be emphasized. On the other hand, there is a need for new innovative treatments, which can postpone NP recurrence and the need of revision surgery, like Omalizumab (anti-IgE) and Mepolizumab (anti-IL-5).

Our findings emphasize the role of a thorough pre-operatively diagnostic evaluation and a targeted long-term medical therapy additional to surgery. Patients should pre-operatively be informed about the marked likelihood of NP recurrence and the need of revision surgery.

## Additional file

**Additional file 1.** Additional information about surgical information, outcome measures, collection of tissue, serum, nasal secretion and statistics.

## Data Availability

The datasets used and analysed during the current study are available from the corresponding author on reasonable request.

## Abbreviations

AERD: aspirin exacerbated respiratory disease
BDL: below detection level
CRSwNP: chronic rhinosinusitis with nasal polyposis
ECP: eosinophilic cationic potein
ESS: endoscopic sinus surgery
IgE: immunoglobulin E
IL-5: interleukin-5
IL-5Rα: interleukin 5 receptor, alpha subunit
IL-18: interleukin-18
IQR: interquartile range
MPO: myeloperoxidase
N: number
ND: not done
NP: nasal polyps
P: probability
SAE-IgE: specific IgE antibodies against *Staphylococcus aureus* enterotoxins
TGF-β1: transforming growth factor beta 1
Th: T helper
