# Supplementary material for: Twelve-year follow-up study after endoscopic sinus surgery in patients with chronic rhinosinusitis with nasal polyposis
Source: Clin Transl Allergy. 2019 Jun 14;9:30. doi: 10.1186/s13601-019-0269-4 (PMC6570859; doi:10.1186/s13601-019-0269-4)
Supplement: Supplementary file 1 — Additional file 1. Additional information about surgical information, outcome measures, collection of tissue, serum, nasal secretion and statistics. [file 13601_2019_269_MOESM1_ESM.docx]

**Additional file 1**

**Surgical procedure**

All patients that underwent surgery between 1998 and 2000 were offered to participate in this study. 47 patients were willing to participate. The same surgeon, C.B., operated all patients under general anesthesia. In our study a standardized surgical procedure was carried out for all patients, during which all sinuses were opened (wide maxillary ostia, complete removal of anterior and posterior ethmoidal cells, sphenoidotomy and frontal recess clearance with irrigation) with conventional instruments and nasal polyps and polypoid mucosa were completely removed. If carrying nasal polyps, the middle turbinate was partially or substantially resected ^1^. Nasal packing was placed and removed 2 days postoperatively and the patient was discharged the second day after surgery. Postoperative treatment consisted of irrigation of the nose with normal saline and vaseline ointment four times a day. Additionally, topical nasal steroid spray (mometasone fuorate or fluticasone propionate) was prescribed for at least three months. Finally, in-office endoscopic debridement was performed weekly for four weeks and monthly for 6 months.

The criteria for revision surgery were based on nasal endoscopy and subjective complaints of the patients after maximal medical treatment. On nasal endoscopy recurrence of nasal polyps was observed and the patients experienced bothersome complaints despite maximal medical treatment.

**Outcome measures**

Symptoms related with CRSwNP (nasal obstruction, rhinorrhea, smell disturbance, sneezing, headache and eye symptoms) were quantified at each contact by the patient as absent (score 0), mild (score 1), moderate (score 2) or severe (score 3). Sum of the individual scores gave a total symptom score between 0 and 18.

One of the primary outcomes was the recurrence rate of nasal polyposis. Pre-operatively and in follow-up endoscopic examination for measurement of polyp size was performed based on the Davos four-point scale (Davos 0: normal mucosa without polyps, Davos 1: small polyps in the middle meatus not reaching below the inferior border of the middle concha, Davos 2: medium-sized polyps below the middle turbinate, Davos 3: large polyps extending below the border of the inferior turbinate).^2^ The total nasal endoscopic polyp score was the sum of the right and left Davos score, ranging from 0 to 6.

The patients were asked if they have undergone ORL-related surgery prior to the ESS in 1998-2000 to identify if this was the first surgery or revision surgery. In the twelve-year follow-up period each surgery and the indication for the surgery was noted to identify the revision surgery rate. Additionally, medical records and surgical protocols were reviewed.

The use of medication for sino-nasal complaints in the last 3 weeks (oral or nasal corticosteroids, asthma medication, antihistamines, antibiotics, nasal saline irrigation) at each time point was assessed.

To evaluate the control of the disease at the three time points we performed the EPOS control test, published in the guidelines of 2012. ^2^ We performed this retrospectively in 2000 and 2006 based on the available data.

To investigate the subjective satisfaction, two different questions were asked. First, the general therapeutic response was questioned as the extent of improvement of symptoms compared to baseline in 1998-2000 and is scored from 1 to 5 (1 = complete relief, 2 = marked relief, 3 = moderate relief, 4 = slight relief, 5 = no relief). Second, the patients were also asked if they would do the initial ESS in 1998-2000 again with the knowledge that they have today.

**Collection of tissue, serum and nasal secretion**

Nasal tissue, namely nasal polyp tissue from CRSwNP patients, was collected during ESS. All samples were immediately processed and stored in aliquots at –80°C until analysis, as previously described ^3^.

Nasal secretions were collected by placing sinus packs (IVALON 4000 plus 3.5x0.9x1.2cm surgical product M-Pact, Eudora, Kan) in both nasal cavities for 5 minutes as described previously ^4^.

Blood was collected by performing a standard venipuncture (serum gel separator tubes Termo Venosafe, Ref VF-106SAS). After allowing the blood to clot for 15-30 minutes, serum was collected after centrifuging at 1600 g for 15 minutes at 4°C. Serum was stored in aliquots at -20°C until further analysis.

Different proteins were measured in all samples: IL-5 (pg/ml, ELISA, Innogenetics), IL-5Rα (pg/ml, ELISA, R&D Systems, Minneapolis, MN, USA), TGF-β1 (pg/ml, ELISA, R&D Systems, Minneapolis, MN, USA), MPO (ng/ml, ELISA, Oxis Research, Immunosource, Zoersel, Belgium) and IL-18 (pg/ml, ELISA, MBL, Naka-ku, Nagoya, Japan). All samples were also assayed for ECP (μg/l), total IgE (kU/l) and specific IgE antibodies (kU/l) by the UniCAP system (Pharmacia Diagnostics, Uppsala, Sweden). The total IgE amount in tissue was further divided in lower than 100 kU/l (RAST grade 1 to 5) or higher then 100 kU/l (RAST grade 6 ^5^). In tissue detectable IL-5 was observed when the amount was above the detection limit of 43.00 pg/ml. IgE against staphylococcus aureus enterotoxins were detectable in tissue when IgE against one of the enterotoxins was measurable (Staphylococcus enterotoxins A, B, C, D, E, TSST).

**Statistics**

The Chi square test or the Fisher’s exact test was applied when comparing two categorical unpaired variables and the Mann-Whitney U test when comparing two continuous unpaired variables. When there were more than two categories for the paired variables (symptoms and nasal endoscopic polyp score), the Bowker’s test for symmetry was used.

To identify possible predictors of NP recurrence and the need of revision surgery after ESS, logistic regression was performed. Odds ratios and their respective 95% confidence interval and P-value were calculated.

‘Surgery-free survival’ following ESS was investigated with Kaplan-Meier analysis based on the exact date of the revision surgery. The Mantel-Cox Log Rank Test was used to compare the ‘surgery-free survival’ curves in categorical variables. Statistical analysis was performed with the SPSS 21.0 software. Statistical significance was assessed using two-tailed tests and was defined as P < 0.05.

TABLES

| **Baseline clinical characteristics** | | | | |
| --- | --- | --- | --- | --- |
|  | **Recurrence**  **(N=30)** | | **No recurrence**  **(N=8)** | **P value** |
| **Clinical characteristics** | | | | |
| Men/women, N/N (%/%) | 18/12 (60/40) | 7/1 (87/13) | | 0.222 |
| Age (y), median (IQR) | 48 (36-54) | 49 (41-68) | | 0.350 |
| Primary/revision ESS in 2000, N/N (%/%) | 14/16 (46.7/53.3) | 5/3 (62.5/37.5) | | 0.693 |
| Total NP score, median (IQR) | 4 (4-6) | 5 (3-5) | | 0.765 |
| Total symptom score, median (IQR) | 9 (7-12) | 9 (8-11) | | 0.928 |
| Comorbidity:  *Allergy, N (%)*  *Asthma, N (%)*  *Samter’s triad, N (%)* |  |  | |  |
|  | 18 (60.0) | 2 (25.0) | | 0.117 |
|  | 12 (40.0) | 3 (37.5) | | 1.000 |
|  | 9 (30.0) | 1 (12.5) | | 0.653 |
| **Tissue biomarkers, median (IQR)** | | | | |
| Tissue IL-5 (pg/ml) | 185.75 (43.00-444.68) | 64.51 (43.00-234.23) | | 0.160 |
| Detectable tissue IL-5, N (%) | 22 (73.3) | 4 (50.0) | | 0.200 |
| Tissue ECP (mg/l) | 10.76 (4.33-16.90) | 2.88 (0.63-16.70) | | 0.137 |
| Tissue IgE (kU/l) | 398.46 (205.39-1213.63) | 600.98 (121.64-1150.09) | | 0.808 |

*Table S1: Baseline clinical characteristics in patients with NP recurrence compared to patients without NP recurrence. N: number; IQR: Interquartile range.*

| Baseline clinical characteristics | | | |
| --- | --- | --- | --- |
|  | **Revision ESS**  **(N=14)** | **No revision ESS**  **(N= 24)** | **P value** |
| **Clinical characteristics** | | | |
| Men/women, N/N (%/%) | 9/5 (64.3/35.7) | 16/8 (66.7/33.3) | 1.000 |
| Age (y), median (IQR) | 38 (34-51) | 51 (41-55) | 0.100 |
| Primary/revision ESS in 2000, N/N (%/%) | 5/9 (35.7/64.3) | 14/10 (58.3/41.7) | 0.179 |
| Total NP score, median (IQR) | 4 (4-6) | 4 (3-5) | 0.273 |
| Total symptom score, median (IQR) | 10 (8-13) | 8 (7-9) | **<0.05** |
| Comorbidity  *Allergy, N (%)*  *Asthma, N (%)*  *Samter’s triad, N (%)* | 11 (78.6)  7 (50.0)  6 (42.9) | 9 (37.5)  8 (33.3)  4 (16.7) | **<0.05**  0.311  0.127 |
| **Tissue biomarkers, median (IQR)** | | | |
| Tissue IL-5 (pg/ml) | 360.84 (103.92-521.84) | 112.94 (43.00-228.88) | **<0.05** |
| Detectable tissue IL-5 | 11 (78.6) | 15 (62.5) | 0.472 |
| Tissue ECP (mg/l) | 12.38 (5.24-19.36) | 8.34 (1.39-15.89) | 0.387 |
| Tissue IgE (kU/l) | 763.60 (365.20-1361.30) | 318.30 (141.33-1073.75) | 0.159 |

*Table S2: Baseline clinical characteristics in patients with revision surgery compared to patients without revision surgery. N: number; IQR: Interquartile range.*

FIGURE

Supplemental figure S1: Medication use at each time point expressed as percentage of study population. The use in the last three weeks of oral, nasal corticosteroids, asthma medication, antihistamines, antibiotics and nasal saline irrigation is shown.


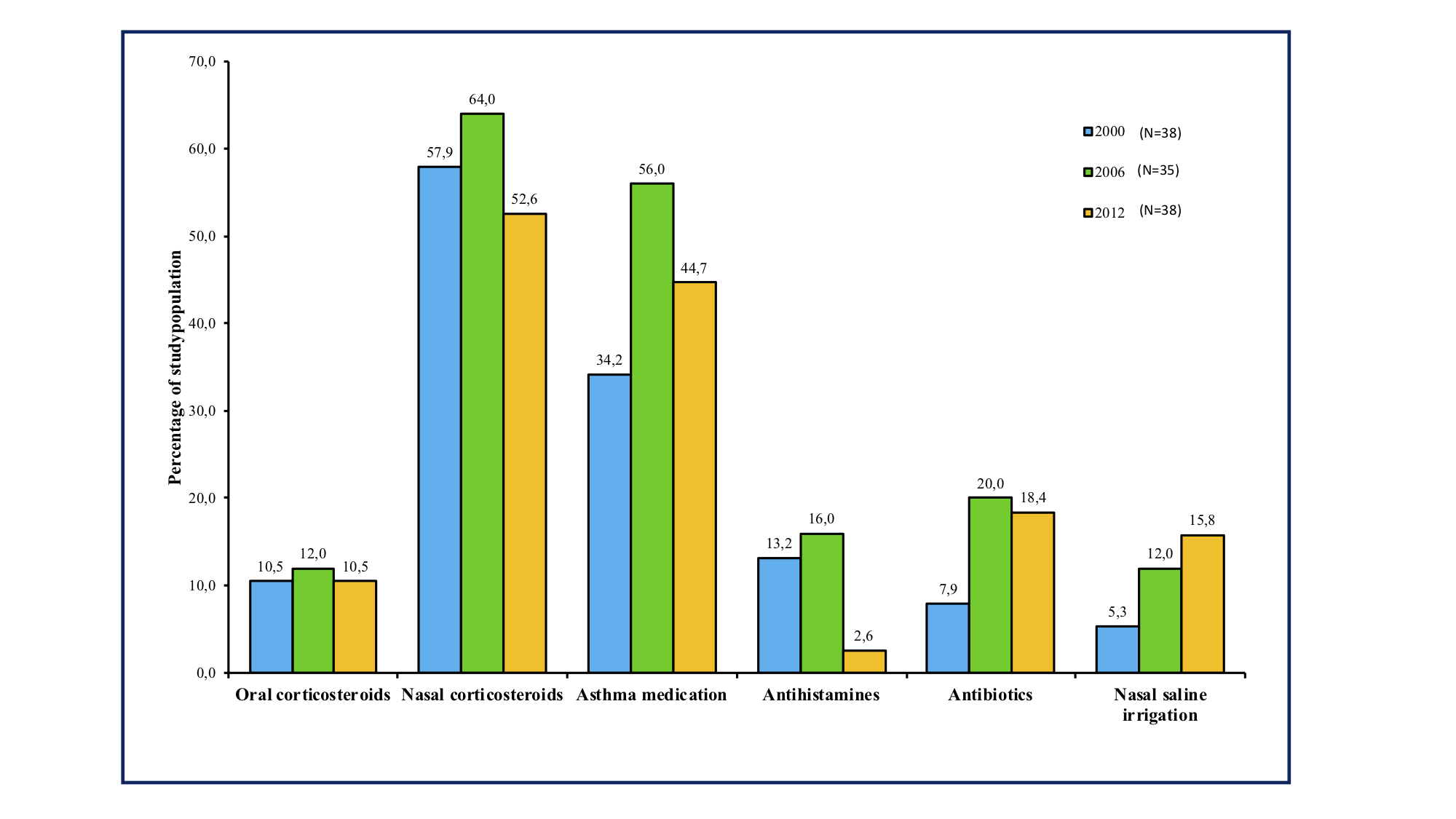


REFERENCES

1. Jankowski R, Pigret D, Decroocq F. Comparison of functional results after ethmoidectomy and nasalization for diffuse and severe nasal polyposis. Acta Otolaryngol 1997;117:601-8.

2. Fokkens WJ, Lund VJ, Mullol J, et al. European Position Paper on Rhinosinusitis and Nasal Polyps 2012. Rhinol Suppl 2012:3 p preceding table of contents, 1-298.

3. Gevaert P, Bachert C, Holtappels G, et al. Enhanced soluble interleukin-5 receptor alpha expression in nasal polyposis. Allergy 2003;58:371-9.

4. Lou ZC, Tang YM, Chen HY, Xiao J. The perforation margin phenotypes and clinical outcome of traumatic tympanic membrane perforation with a Gelfoam patch: our experience from a retrospective study of seventy-four patients. Clinical otolaryngology : official journal of ENT-UK ; official journal of Netherlands Society for Oto-Rhino-Laryngology & Cervico-Facial Surgery 2015;40:389-92.

5. Hamilton RG, Williams PB. Human IgE antibody serology: a primer for the practicing North American allergist/immunologist. J Allergy Clin Immunol 2010;126:33-8.
